# Improving the quality of cataract surgery

**Published:** 2014

**Authors:** Robert Lindfield

**Affiliations:** Lecturer: International Centre for Eye Health and Advisor: ORBIS, London, UK. robert.Lindfield@Lshtm.ac.uk

**Figure:**
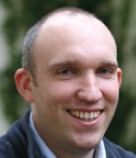
Robert Lindfield

Quality is a difficult concept to describe and there is no clear definition. People have different views of what quality means and describe it in different ways.

Take the example of a trip by taxi. How would you describe the quality of the trip? Do you base your description on the politeness of the driver or the luxury of the vehicle? Or was it that you arrived at your destination safely? If you asked the driver, how would they describe the quality of the same trip? They might describe quality in terms of a trip without breaking down or being held up in traffic. There are clearly several different ways in which quality can be measured or described, and the ones that interest you will depend on who you are.

Equally, there are many different ways of describing the quality of cataract surgery (some which will be of more interest to patients and others which will be of interest mainly to surgeons). The quality of cataract surgery can be described in terms of:

- clinical effectiveness
- patient safety
- patient experience
- cost-effectiveness
- equity (equal access).

These are also referred to as ‘domains’ of quality. For the purpose of this article we will focus on the first three.

## Clinical effectiveness

Clinical effectiveness measures the success of the surgical procedure; i.e., is the patient's vision good?

If you ask most surgeons to describe the quality of an operation, they will talk about the outcome of the operation (the visual acuity). In other words, they define quality as clinical effectiveness. Other indicators of clinical effectiveness include the patient's clinical details before, during and after surgery. Taken together, these aspects of care describe clinical quality.

### Visual acuity

Visual acuity (VA) is the most common clinical measure of the quality of cataract surgery. It is how we describe and measure the success of surgery and it is therefore critical that it is measured well. Unfortunately, VA is often measured inaccurately, frequently by junior, or untrained, staff. Whilst the poor measurement of VA does not directly impact on the patient, it means that surgeons and the hospital are unable to assess the clinical effectiveness of their care. A high rate of poor outcomes after cataract surgery is a cause for concern and should lead to investigation of potential reasons. A low rate of poor VA is cause for celebration but it should not be taken for granted.

**Figure:**
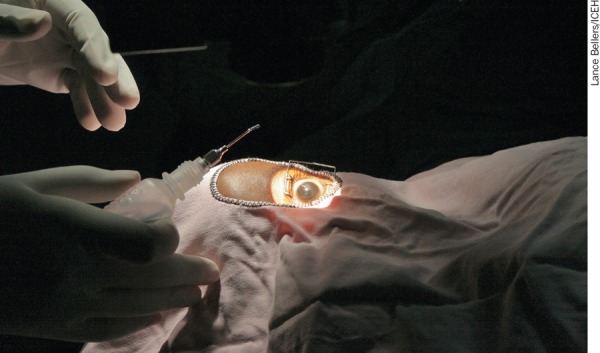
Patient safety is an important component of quality

Measurement of VA must be standardised and systematic. All staff members should measure VA in the same way, using the same steps (see article on page 16), and they should record the visual acuity in the same way in the case notes. Therefore, a patient's recorded VA should be the same, regardless of which staff member measured it. The following are minimum standards for VA measurement.

- All patients undergoing cataract surgery should have VA measured pre- and post-operatively. If someone is an in-patient, VA should be measured every day. If someone is a day patient, it should be measured at every follow-up visit.
- VA should be measured using a standard chart with clear letters on a white background (preferably back-lit), from a standard distance.
- Pinhole acuity should be measured for every patient if possible.

### Benchmarking

The World Health Organization (WHO) has a set of recommendations^1^ for the percentage of operations with good, borderline or poor visual outcome following surgery. Unfortunately, the guidelines do not specify when after surgery this should be measured. More useful than these guidelines is the concept of benchmarking.

Benchmarking allows hospitals to compare their current performance against their past performance and set targets for how much they want to improve or change their performance. It is also possible to use benchmarking to compare their performance with that of other similar hospitals.

Benchmarking involves recording the VA following a set number of operations at specific times (e.g. at discharge, first or second follow-up), and determining the proportion of operations that achieve a certain VA at that point (e.g. a VA of 6/9 at first follow-up). This proportion is then used as a baseline or benchmark to judge whether outcomes have improved at a later point in time. Recording the VA of 200 operations is generally enough to provide a good baseline.

**Using benchmarking to improve performance**

1. To create a baseline measurement, measure and record VA following 200 consecutive cataract operations at one or more of the following points: at discharge, at first follow-up, and/or at second follow-up.
2. Carry out any planned quality improvement activities.
3. Once the activities have become established and consistent practice, measure and record VA after another 200 consecutive cataract operations **at the same point(s)** as in Step 1 above.
4. Repeat every month or every quarter to monitor progress.

**Figure:**
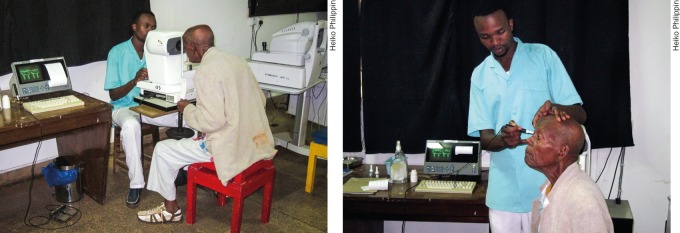
Biometry is essential to calculate the power of the intraocular lens and to achieve good visual outcome

It is also possible to set targets for improvement based on the baseline. The hospital might decide that it wants the proportion of patients achieving 6/9 vision at first follow-up to increase by a given percentage over the following six months and put measures in place to achieve this.

### Biometry

Biometry, the measurement of the power of the intraocular lens pre-operatively, is as critical as good surgical technique in ensuring good visual outcome.

Where clinically possible, every hospital should routinely use biometry for patients undergoing cataract surgery. If biometry is not available, then the hospital should consider whether cataract surgery should continue, as the risk of sub-optimal VA after the operation is very high.

### Complications

The other important marker of clinical quality is the complication rate – the number of complications per 100 operations. There are numerous complications of cataract surgery and it is important that these are recorded in the case notes – both honestly and in detail.

Every surgeon has complications. Recording, understanding and addressing complications is an important part of ensuring the quality of cataract surgery in an individual and in an institution.

All other relevant clinical details must be recorded systematically for every cataract operation performed (see panel opposite). Without this information it is impossible for the hospital or surgeon to understand their case load, identify issues and make changes.

**Note**: The complication rate can be used in benchmarking in a similar way as VA (see the panel on page 9).

## Patient safety

More than 1 in 10 patients in hospital experience a harmful incident unrelated to their condition. These incidents are largely preventable and recognition of the scale of the problem has led to the WHO starting a patient safety programme. The prospect of legal action and costly payouts has led many hospitals, even in low-income countries, to take patient safety seriously.

Patient safety in cataract surgery is critical. Harm can occur at any time during the patient pathway, from tripping over in the outpatient department to endophthalmitis from poorly sterilised equipment. Most incidents are preventable given a culture of patient safety within the hospital and motivated staff.

There are two ways of approaching patient safety: retrospectively, where an adverse incident is investigated to find out the cause, and prospectively, where harm is prevented.

Retrospective incident investigations are critical; however, they must be conducted in a systematic and fair way. There is a concept called ‘no blame’ in which the hospital recognises that, usually, there is a series of errors or mistakes that led to the patient safety incident. The purpose of the investigation is to find out what errors occurred and the main causes of these errors. ‘No blame’ does not mean ‘no responsibility’, however, and it is important that, if someone is culpable, they are disciplined appropriately. Every hospital should have a policy of investigating patient safety incidents with clear guidelines about who is responsible and how they should proceed.

Prevention is better than cure, so ideally a hospital should try and implement as many preventative activities as possible. The WHO has published guidance to help hospitals in low-income countries ensure that their patients are safe.^2^

Most patient safety incidents occur in the operating theatre, thus ensuring the patient does not come to harm in this setting is important. Whilst every hospital recognises the importance of sterilising instruments or wearing gloves, how many think that checking the patient's name, the strength of the intra-ocular lens to be inserted or which eye it is going to be put in, is important?

Most surgeons have experienced operating on the wrong patient, inserting the wrong IOL or operating on the wrong eye. To help prevent such incidents, the UK's National Health Service (NHS) has adapted the WHO surgical safety checklist for cataract surgery. A copy is available online^3^ and its use for all cataract operations is highly recommended.

It is widely recognised that the culture of patient safety within a hospital determines how likely patients are to be harmed. Culture describes the attitude of management and staff to patients and their care.

A hospital with a good culture of patient safety has a range of characteristics:

- patient-centred care, where patients are treated with respect
- a good environment with all the necessary equipment
- a strong clinical team that works well together
- the necessary support from management.

There are tools available to assess the culture of patient safety within a hospital and to identify areas that require further strengthening.

## Patient experience

In the example earlier you were asked to describe the quality of a taxi ride. Asking users to describe their experiences is commonly used by service providers outside of healthcare to assess the quality of their service. Hospitals have started measuring patient experience so they can measure the quality received by their clients, the patients.

**How to measure quality**

To know the quality of its cataract surgical service, each hospital must measure quality. There are a variety of tools available to collect and analyse clinical and patient safety data. Collecting patient experience data is a bit more complicated, but simple measures such as a suggestion box or patient discussion groups (where patients come together after surgery with a counsellor and discuss their experience) can be put in place to allow patients to provide feedback that the hospital can act on.

**Collecting data on quality**

The following are the resources required to collect data on quality:

1. tools (computer and software) to enter data on quality
2. someone to collect and enter the data
3. someone to analyse and interpret the results
4. a forum (meeting) to discuss the results with all staff (everyone has a role in quality)
5. strong leadership to make decisions (sometimes difficult) and push through changes.

**Creating change**

Changing human behaviour is always difficult. Staff have done the same thing for many years and getting them to change is often challenging. To improve quality, however, change is necessary. Better never stops!

**Approaches to change**

**Communication**

Open and honest discussion with staff members about the issues and how to address them effectively is an important first step to improving quality. For example, what prevents staff from measuring visual acuity (VA) accurately? Is it the equipment they have, or that they are expected to check the VA of 45 patients in 30 minutes, or that they don't ever see anyone checking what they've written in the notes? Staff members often have ideas about how things could be improved that are practical and contextual.

**Trial and error**

Rarely is major change either possible or effective. Change usually occurs in stepwise increments. Making small changes, and checking whether they work, or not, is an important part of quality improvement. For example, you might change the way that patients have their VA assessed so that it is more manageable for the staff members concerned. You would then continue to discuss the effectiveness of this change with staff.

**Embedding change**

Often, when new ideas are first brought forward, some people embrace them, but there is a tendency for people to revert to their old ways of working. It is critical that any quality improvement activities are actively monitored and supported, not just in the short term.

In the past, studies have measured patient satisfaction with care they receive. Unfortunately, most patients in low-income countries report very high levels of satisfaction even when they have not received good care. This appears to be the result of patients not wishing to speak badly about the hospital or clinical staff, but leads to an artificially high satisfaction level. A second issue with patient satisfaction is that the presence or absence of satisfaction is of limited use to a hospital. Being satisfied or dissatisfied tells the hospital nothing about their service or how to improve it.

These issues with patient satisfaction have led to the use of patient experience as a measure of quality. Patient experience describes the patient's views about their experience of different aspects of care; for example, was the hospital clean? What did the staff tell you to do after your operation? These can be checked internally and, if enough patients report specific issues, they can be acted on.

## Conclusion

We are keen to conduct as much cataract surgery as possible; however, evidence suggests that visual outcome is frequently sub-optimal in many low-income settings. Improving visual outcome is complicated and requires a holistic approach to patient care involving a focus on clinical effectiveness, patient safety and patient experience.

As a minimum, every hospital should be collecting clinical effectiveness data on every cataract operation. It should also have in place patient safety protocols like the WHO surgical safety checklist as well as procedures describing what to do if a patient is harmed. Finally, collecting information about patient experience is important to make sure that patients receive the care they need.

Making changes in the cataract surgery service is difficult and requires good communication and perseverance. Change does not happen overnight and needs to be supported and encouraged. This requires strong leadership.

## Clinical Quality Group

The International Centre for Eye Health is launching a Clinical Quality Group that aims to provide a forum for clinical staff who want to improve the quality of their service. There will be a regular newsletter and a chance to link with other clinicians who are focusing on quality.

To take part, email Robert Lindfield (robert.lindfield@Lshtm.ac.uk).

**Figure:**
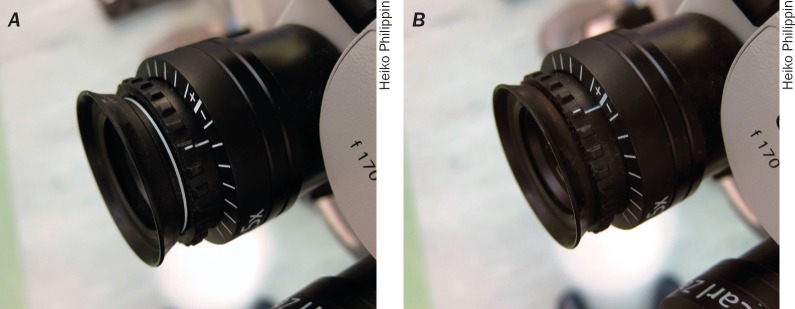
Preparing for surgery also includes the small details. If the microscope is not set up correctly (A). it will compromise the surgeon's view during the procedure. B shows the correct setup.
